# Impact of Curing Time and Temperature on Bond Performance of Epoxy Resin Adhesives for Steel Bridge Decks

**DOI:** 10.3390/polym17081018

**Published:** 2025-04-09

**Authors:** Chuanbin Fan, Huanyong Chen, Feng Lin, Weixiong Li, Xuetang Xiong, Bo Chen, Huayang Yu

**Affiliations:** 1 Shenzhen-Zhongshan Link Administration Center, Guangzhou 510000, China; 18688266279@163.com; 2 Guangdong Guanyue Highway & Bridge Co., Ltd., Guangzhou 511450, China; 13042080126@163.com (H.C.); 13502335896@163.com (F.L.); 3 Xiaoning Institute of Roadway Engineering, Guangzhou 510006, China; 201810101689@mail.scut.edu.cn; 4 School of Civil Engineering and Transportation, Foshan University, Foshan 528225, China; xuetangxiong@163.com; 5 School of Civil Engineering and Transportation, South China University of Technology, Guangzhou 510006, China; huayangyu@scut.edu.cn

**Keywords:** steel bridge deck, epoxy resin, bonding performance, curing time, curing temperature, tensile strength, tack-free time, construction workability

## Abstract

The bonding performance of epoxy resin adhesives plays a critical role in ensuring interlayer adhesion and long-term durability in steel bridge deck pavements. However, the construction environment temperature and curing time significantly influence the bonding properties of epoxy resin adhesives. To address this issue, systematic evaluations of the bonding performance and tack-free time of epoxy resin adhesives were conducted. The results demonstrate that under identical curing durations, the tensile bond strength between the epoxy resin bonding layer and steel plate increases with higher curing temperatures. Similarly, at constant curing temperatures, extended curing times lead to improved tensile strength. Both higher temperatures and longer curing durations reduce the tack-free time of the epoxy resin adhesive. Under natural outdoor curing conditions, the epoxy resin adhesive achieves design requirements for both tensile strength and tack-free time after 48 h of curing, ensuring optimal interlayer bonding and workability. Conversely, prolonged curing beyond 72 h results in significantly reduced bonding strength while maintaining acceptable tack-free time. For ambient temperature conditions, the optimal curing duration for epoxy resin adhesive is determined to be 48~72 h, balancing both bonding performance and construction requirements (preventing adhesion to construction equipment). This research offers technical guidance for the field construction of epoxy pavement on steel bridge decks by establishing optimal curing protocols for epoxy resin adhesives to ensure reliable bonding performance and construction workability.

## 1. Introduction

Steel bridge deck pavements are an essential component of modern bridge infrastructure, where the interlayer bonding between the steel surface and the paving materials is critical to the overall performance and durability of the structure [1,2,3]. The adhesion of the overlay material to the steel deck directly influences the long-term stability, fatigue resistance, and protection of the bridge surface [4,5]. Over the years, epoxy-resin-based bonding layers have emerged as a promising solution due to their superior adhesive strength and resistance to environmental degradation [6,7]. However, the performance of these bonding systems under various curing conditions, environmental exposures, and operational stresses remains an area of active research, aiming to optimize the application of epoxy resin adhesives for steel bridge decks.

The use of epoxy resin as a bonding layer in steel bridge deck pavements has gained increasing attention over the past few decades due to its exceptional adhesion properties and durability [8,9,10]. Epoxy resin is known for its strong bond to metal surfaces, high chemical resistance, and excellent mechanical properties, making it ideal for steel deck pavements exposed to harsh conditions [11,12,13]. Numerous studies have explored the effects of various factors on the bonding performance of epoxy resin adhesives, focusing primarily on curing conditions, environmental exposure, and material composition [14,15,16,17].

One of the most critical factors influencing the performance of epoxy resin bonding layers is curing temperature and curing time [18,19]. Research has shown that curing at elevated temperatures can significantly enhance the adhesive strength of epoxy resins due to the accelerated polymerization and crosslinking of the resin molecules [9]. For instance, studies by Zhang et al. indicated that curing temperatures in the range of 50~60 °C lead to significant improvements in bond strength compared to ambient temperature curing, which is commonly used in field applications [20]. However, excessively high curing temperatures (>80 °C) may lead to thermal degradation of the resin or cause it to become brittle, reducing the long-term durability of the bond. On the other hand, curing time also plays an important role: longer curing times generally lead to stronger bonds, but excessively prolonged curing (>14 days) can cause the epoxy resin to harden too much, diminishing its flexibility and crack resistance [21,22].

In a study by Li et al. [23], the effects of different curing times were examined at a specific curing temperature, revealing that bond strength continued to increase with curing times up to 48 h, beyond which no significant improvements were observed. This indicates that there is an optimal curing time, beyond which additional curing may not contribute substantially to bond strength but could negatively affect the material properties [24,25]. Thus, determining the optimal curing time and temperature for epoxy resin adhesives is crucial for achieving the best balance between bond strength, flexibility, and long-term durability.

Epoxy resins, while highly durable under controlled laboratory conditions, often face degradation when exposed to harsh environmental factors, especially UV radiation, moisture, and temperature fluctuations [21,26,27]. These environmental stresses can weaken the bond strength of the adhesive layer, leading to delamination or the premature failure of the pavement system. Wang et al. explored the impact of long-term outdoor exposure on epoxy resin bonding layers, showing that UV radiation and moisture penetration lead to a significant reduction in bond strength over time [13]. UV radiation accelerates the breakdown of the resin’s molecular structure, causing surface degradation and reduced adhesion properties [22,28]. Moisture, on the other hand, can seep into the epoxy resin layer, weakening the bond between the resin and the steel deck [29].

In contrast, studies by Chen et al. suggested that the addition of UV stabilizers and moisture-resistant additives to the epoxy resin could significantly improve its resistance to environmental degradation [30]. These modified resins showed enhanced bond strength even after prolonged exposure to UV light and moisture, indicating that proper formulation adjustments can greatly enhance the performance of epoxy resin bonding layers in real-world conditions [21]. Protective coatings or sealants, as suggested by several researchers, are another potential solution to mitigate the effects of environmental exposure on epoxy resin performance.

To further improve the performance of epoxy resin bonding layers, researchers have also explored the use of modified epoxy resins [7,24,25,30]. One notable modification is the incorporation of rubber or polymer additives to improve the flexibility and impact resistance of the resin [31].

An innovative approach in recent studies is the integration of epoxy resin adhesives with epoxy asphalt concrete [32]. This combination offers a dual benefit of high adhesive strength and improved performance of the overlay material. Epoxy asphalt concrete, when combined with an epoxy resin bonding layer, forms a cohesive bond that enhances the overall structural integrity of the pavement system [33].

The integration of epoxy resin with asphalt concrete is particularly advantageous in cases where rapid construction is necessary, such as in highway and bridge repairs [34]. This method offers the possibility of reducing maintenance costs while providing a longer-lasting and more resilient pavement system [35]. Additionally, this integrated approach reduces the risk of water infiltration and environmental degradation, which are common causes of pavement failure in steel bridge decks [36,37,38].

The long-term durability of epoxy resin bonding layers is a major concern in steel bridge deck pavements [39]. Several studies have investigated the fatigue resistance and creep behavior of epoxy resin adhesives under repeated loading and varying environmental conditions [40,41,42]. A study by Zhang et al. found that epoxy resin adhesives maintained their bonding strength even under cyclic loading, which is commonly experienced by bridge decks [43]. However, the resin’s ability to resist creep deformation—gradual changes in shape under constant load—was found to decrease over time under high-temperature conditions. This highlights the importance of not only optimizing curing conditions, but also ensuring that the epoxy resin can withstand the long-term stresses imposed on the pavement structure [44].

While significant advancements have been made in understanding the performance of epoxy resin bonding layers for steel bridge decks, challenges remain regarding the optimization of curing conditions, environmental resistance, and material properties to ensure long-term durability [5,45,46].

Building on the progress of previous research, this study aims to investigate the impact of curing temperature and curing time on the bond strength of epoxy resin adhesives in steel bridge deck pavements. Specifically, this research will focus on identifying the optimal curing conditions that maximize bond strength without compromising the flexibility or long-term performance of the resin. Additionally, the tack-free time of the epoxy resin adhesive will be analyzed to obtain the optimal application time for achieving the best construction workability. Finally, this research will determine optimal curing durations for the epoxy resin bonding layer under various construction temperatures to ensure the durable performance of steel bridge deck pavements.

## 2. Bonding Principle of Epoxy Resin Adhesives

Epoxy resin adhesives achieve bonding through dual mechanisms: mechanical interlocking and chemical adhesion [47,48]. The resin penetrates the surface irregularities of steel decks, creating mechanical anchorage, while its reactive epoxide groups form chemical bonds with steel substrates. This combination ensures strong, durable adhesion, which is critical for bridge applications.

**Figure 1:**
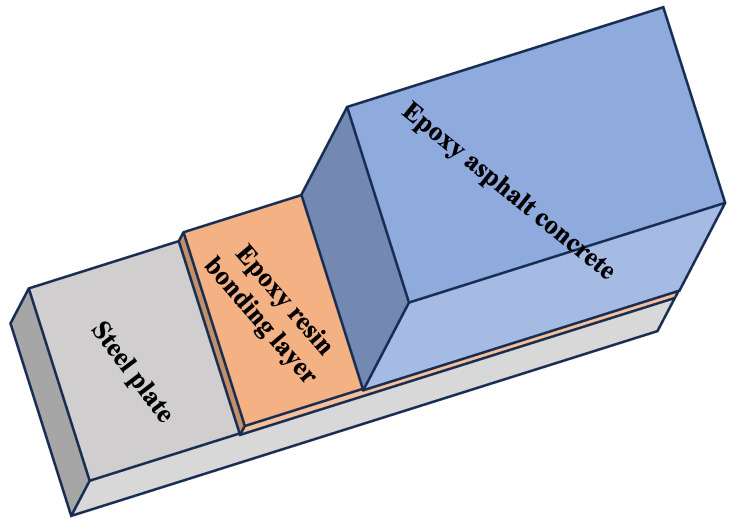
Composite structure of steel plate, epoxy resin bonding layer, and epoxy asphalt concrete.

When bonding steel to epoxy asphalt mixtures, the adhesive interacts through (1) polar attraction between epoxy groups and asphalt components and (2) mechanical embedding within the aggregate matrix, as shown in Figure 1. The curing process enhances these bonds, developing an interface resistant to structural stresses.

Bonding performance depends on three key factors: proper curing (temperature/time optimization), surface preparation (cleaning/roughening), and material formulation (additives for flexibility and environmental resistance). These parameters collectively determine the adhesive’s long-term durability under operational conditions.

The subsequent study will focus on investigating the effects of curing temperature and time on the bonding performance of epoxy resin adhesives, aiming to establish optimal construction parameters for epoxy resin adhesive applications.

## 3. Properties of Experimental Raw Materials

### 3.1. Epoxy Resin Adhesive

The waterproof bonding layer and adhesive layer use KD-HYP epoxy resin adhesive. The epoxy resin adhesive consists of two components: the base resin and the hardener. Its basic physical properties and technical specifications are shown in Table 1 and Table 2.

Testing the properties of both the epoxy resin (base) and hardener (curing agent) is crucial for ensuring the adhesive’s performance in demanding applications, such as steel bridge deck pavements. The resin and hardener must be compatible and react properly to form a strong, durable cross-linked network upon curing. Inadequate testing of these components can lead to incomplete curing, weak adhesion, and poor mechanical properties, compromising the bond strength and longevity of the adhesive layer.

The temperature of the base agent and curing agent before mixing should be controlled at 25 ± 2 °C using a dedicated constant-temperature room. The mixture should be electrically stirred for 3 min at a 50:50 weight ratio. The properties of the cured epoxy resin adhesive are presented in Table 3.

The curing process forms a cross-linked polymer network, with bond strength, flexibility, and durability dependent on curing conditions and resin–hardener compatibility. Testing the cured epoxy resin is crucial to ensure it meets the required mechanical properties, such as tensile and shear strength, and resists environmental factors like moisture, UV exposure, and temperature fluctuations. Verifying these properties ensures the adhesive’s long-term reliability and effectiveness, particularly in steel bridge deck pavements, where strong, durable bonds are essential for structural integrity.

### 3.2. Epoxy Resin Binder in Epoxy Asphalt Concrete

Epoxy resin binder, a key material for hot-mixed epoxy asphalt concrete pavement layers, consists of the epoxy resin base agent and curing agent. The KD-BEP epoxy resin binder is used in this study. The physical properties and technical indicators of the base agent and curing agent are detailed in Table 4 and Table 5. The properties of the cured epoxy resin adhesive are shown in Table 6.

In epoxy asphalt concrete, the resin enhances the bonding strength between the asphalt binder and aggregate, significantly improving the pavement’s overall durability and performance. It acts as a strong adhesive, resisting cracking, fatigue, and moisture damage. Thorough testing of the base agent and curing agent’s physical properties, such as viscosity, tensile strength, and flexibility, is essential to ensure they meet the necessary criteria after curing. This ensures the cured resin can effectively bond with both the steel deck and asphalt mixture, providing long-term stability and resistance to environmental stresses like temperature fluctuations and moisture.

### 3.3. Asphalt Binder in Epoxy Asphalt Concrete

The asphalt used is Grade A 70# matrix asphalt. The matrix asphalt meets the technical indicators stated in Table 7.

The asphalt binder serves as the glue that holds the aggregates together and plays a key role in determining the pavement’s resistance to fatigue, moisture damage, and thermal cracking. By evaluating key properties such as viscosity, penetration, softening point, and aging resistance, these tests ensure that the asphalt meets the necessary standards for bonding strength and long-term stability. Proper performance testing of the asphalt binder ensures that the epoxy asphalt concrete can withstand the harsh environmental and operational conditions typical of bridge decks, enhancing the pavement’s longevity and reliability.

### 3.4. Aggregates in Epoxy Asphalt Concrete

Coarse aggregate is made of high-quality crushed stone aggregates such as basalt, diabase, and andesite, with a particle size greater than 2.36 mm. The particle shape is approximately cubic. The physical and mechanical properties of the coarse aggregate must meet the requirements in Table 8, and the aggregate particle size specifications should be selected according to the requirements in Table 8.

The quality of coarse aggregate directly impacts the bonding between the asphalt binder and the aggregate, as well as the mixture’s resistance to cracking, rutting, and wear. Key properties such as particle size distribution, surface texture, absorption, and strength must be tested to ensure that the aggregate provides a stable, durable framework for the mixture. Properly tested coarse aggregate helps to optimize the performance of the epoxy asphalt mixture, ensuring it can withstand the mechanical stresses, thermal cycles, and environmental exposure typical in infrastructure applications like bridge decks. This contributes to the long-term serviceability and safety of the pavement.

Fine aggregate is made of high-quality crushed sand with a particle size between 2.36 mm and 0.075 mm, using materials such as basalt, diabase, and andesite. It must be free of impurities or other harmful substances. The technical requirements are shown in Table 9.

Fine aggregate contributes to the overall texture and stability of the asphalt, affecting the bonding between the asphalt binder and the coarse aggregate. Key tests on properties like particle size distribution, gradation, and cleanliness help ensure proper mixing and optimal performance. Well-tested fine aggregates improve the mixture’s resistance to rutting, cracking, and moisture damage, thus enhancing the long-term durability and functionality of the epoxy asphalt pavement, especially in environments exposed to heavy traffic and variable weather conditions.

Mineral filler should be made from hydrophobic stones, such as limestone or basic igneous rocks, and ground into mineral powder. It should not contain soil impurities or agglomerates, and must be dry and clean, able to flow freely from the mineral powder storage bin. Its quality must meet the technical requirements in Table 10.

Mineral powder plays an important role in enhancing the properties of asphalt mixtures, including improving workability, increasing stiffness, and enhancing adhesion between the binder and aggregate. It helps to optimize the rheological properties of the asphalt binder, making it more resistant to deformation under heavy traffic loads. Additionally, mineral powder can fill voids within the mix, improving its density and compactability, and contributing to better resistance to moisture damage and fatigue. It also enhances the chemical interaction between the binder and aggregate, leading to improved long-term durability and performance, particularly in epoxy asphalt mixtures used in harsh environmental conditions.

### 3.5. Epoxy Asphalt Concrete

**Figure 2:**
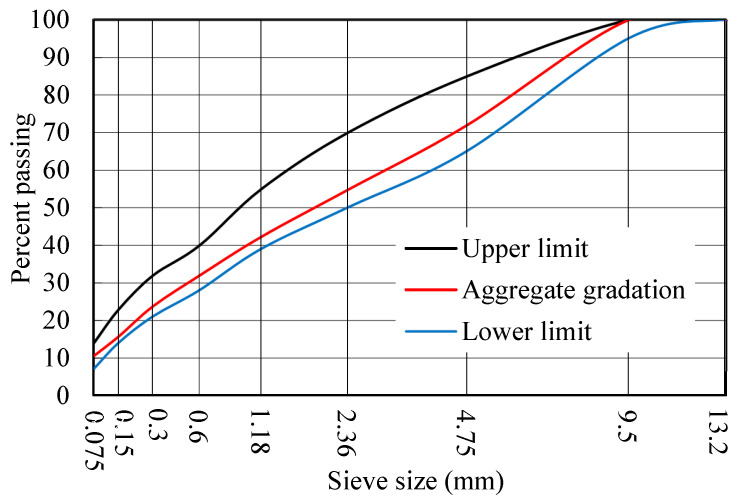
Aggregate gradation of epoxy asphalt mixture (EAC-10).

The aggregate gradation of hot-mixed epoxy asphalt concrete (EAC-10) is shown in Figure 2, with an oil–stone ratio of 6.5%. The performance of the epoxy asphalt concrete is shown in Table 11.

The mix proportion of epoxy asphalt mixture is crucial for achieving optimal performance, as it directly influences the workability, strength, and durability of the pavement. Properly designed proportions ensure that the binder, aggregates, and additives are well-balanced to provide adequate bonding, resistance to deformation, and long-term stability under varying environmental and traffic conditions. The performance testing of the mixture, including tests for rheological properties, stiffness, fatigue resistance, and moisture susceptibility, is essential to verify that the mix meets the required standards for durability and safety. These tests help assess how well the mixture will perform in real-world conditions, ensuring that the epoxy asphalt pavement will maintain its integrity and function over time, even under heavy loads and extreme weather.

## 4. Experimental Program of Epoxy Resin Adhesives

The bonding performance of epoxy resin adhesives for steel bridge decks is significantly influenced by curing time and temperature. These factors play a crucial role in determining the adhesive’s ability to form a strong and durable bond between the steel deck and the epoxy asphalt overlay. In particular, the curing time required for the adhesive to reach sufficient strength is a critical parameter that impacts the overall bonding effectiveness. Additionally, the temperature during curing can affect the crosslinking rate of the epoxy resin, influencing its final mechanical properties.

Moreover, it has been observed that construction machinery can experience severe adhesive wheel drag if the adhesive layer is not fully cured before the application of the epoxy asphalt mixture. This issue arises because the adhesive has not yet reached a sufficient level of dryness or strength to withstand the pressure and movement of construction equipment. Therefore, it is imperative to determine the dry-to-touch time of the epoxy resin adhesive, which indicates the point at which it is safe to begin the overlay construction without risking mechanical damage to the adhesive layer.

To address these concerns, this study focuses on the experimental evaluation of the bonding performance of epoxy resin adhesive under varying curing times and temperatures. Furthermore, the drying and setting times of the adhesive are examined to establish optimal curing conditions for successful pavement construction. The results will provide valuable insights into the time–temperature sensitivity of epoxy resin adhesives and help optimize construction processes to ensure both the durability and efficiency of steel bridge deck pavement installations.

### 4.1. Bonding Performance of Epoxy Resin Adhesives—Experimental Design

After the application of traditional epoxy resin adhesives, curing is required before the adhesive reaches a solid state, allowing the epoxy asphalt mixture to be laid. However, the curing time must not be excessively long, as prolonged curing may lead to a decrease in interlayer bonding performance, which in turn affects the overall stability of the pavement layer. Therefore, the standard construction procedure for epoxy resin adhesives typically involves a one-day curing period before the construction of epoxy asphalt concrete, with a full construction cycle lasting three days. This process imposes strict requirements.

Given the large-scale paving of steel bridge decks in the Shenzhen–Zhongshan Corridor, which is subjected to high humidity, high salinity, and complex weather conditions, using conventional construction methods poses certain risks. Therefore, it is necessary to investigate the bonding performance of epoxy resin adhesives under different curing times.

After the application of the epoxy resin adhesive, curing is required before the adhesive reaches a solid state, allowing the epoxy asphalt mixture to be laid. However, the large-scale paving of steel bridge decks in the Shenzhen–Zhongshan Corridor is subject to high humidity, high salinity, and complex and variable weather conditions, which pose certain risks when using conventional construction methods. Therefore, specimens comprising a “steel plate + epoxy zinc-rich primer + waterproof bonding layer” were exposed to natural environmental conditions. The evaluation of the bonding performance under different curing times was conducted during the traditional paving period in Guangdong Province, i.e., from October to December.

The cohesive strength of the bonding material is primarily evaluated through pull-off tests, which include direct pull-off tests on the bonding layer, pull-off tests on the composite structure of the formed paving layer, and pull-off tests on the structure of the actual construction paving layer. The first two tests are used to directly assess the bond strength between the bonding layer and the steel plate (including the corrosion-resistant coating) and to evaluate the overall bonding performance of the epoxy asphalt concrete and steel plate composite structure. The latter test reflects and verifies the tensile performance of the bonding material within the paving layer.

This study primarily focuses on evaluating the bond strength between the bonding layer and the steel plate (including the corrosion-resistant coating) and the overall bonding performance of the epoxy asphalt concrete and steel plate composite structure under different curing times of the epoxy resin adhesive.

The pull-off test procedure is based on the standards outlined in “Paints and Varnishes: Pull-Off Test for Adhesion” (GB/T 5210-2006) [56] and “Determination of Adhesion Strength Between Vulcanized Rubber or Thermoplastics and Metals” (GB/T 11211-2009) [57], as well as other research projects on bonding layers for steel bridge deck paving. The design of the pull-off test method follows these guidelines.

The steel plate used in the experiment has the same material and thickness as that used in the actual bridge, with Q345 steel plates measuring 300 ×300 × 16 mm. Prior to the test, the plates undergo sandblasting and corrosion-resistant coating treatments, replicating the actual construction process. After sandblasting, the roughness of the steel plate reaches 80~150 μm, and the cleanliness is equivalent to Sa3.0 level. The paving materials used in the experiment are also the same as those in the actual construction process.

Steel plates of the same material and thickness as the actual bridge deck are selected for sandblasting and coating with a corrosion-resistant layer. The thickness of the epoxy zinc-rich paint film is measured to be between 80 and 120 μm, and the curing period lasts at least 15 days. The epoxy resin adhesive is mixed at a 1:1 ratio of base resin and hardener, using mechanical stirring at a speed of 500 rpm for 3 min at room temperature (25 °C) to ensure the thorough blending of components A and B. The test is conducted at four different curing temperatures: 15 °C, 25 °C, 40 °C, and 60 °C. These temperatures are chosen to reflect the typical climate variations in the coastal region around Shenzhen, where the epoxy asphalt concrete pavement is intended to be applied. This range includes both moderate and elevated temperatures, allowing us to evaluate the performance and curing behavior of the epoxy resin adhesive under diverse environmental conditions. The curing times are set at 1 day, 2 days, 3 days, and 4 days, respectively, in order to assess the bonding strength and its variation with curing time on the steel bridge deck, as shown in Table 12.

**Figure 3:**
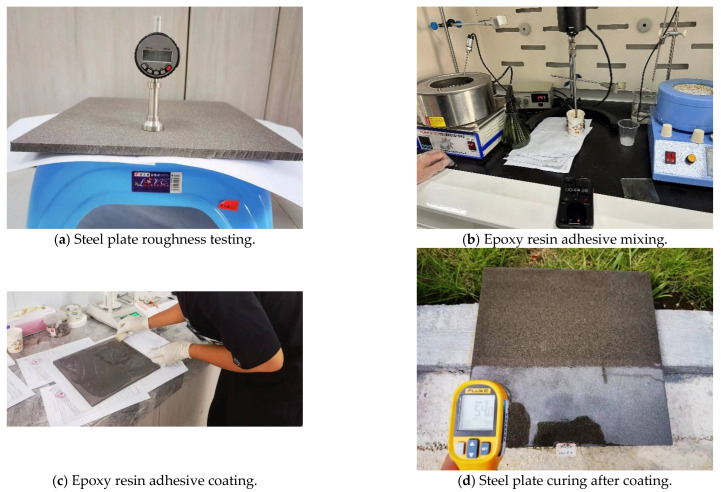

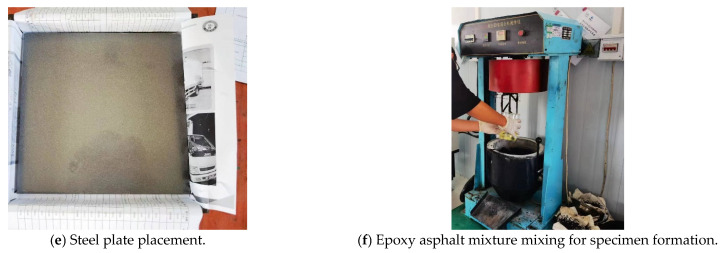
Flowchart of composite specimen preparation.

The epoxy resin adhesive is applied according to the specified amount (0.6 kg/m^2^). After curing, the hot-mix epoxy asphalt concrete paving layer is formed on the steel plate using the roller compaction method, resulting in a composite specimen. The curing times are set to 0 h, 24 h, 48 h, 72 h, and 96 h, with outdoor natural curing temperatures ranging between 25 °C and 35 °C. After curing, pull-off tests are conducted to evaluate the overall bonding performance of the epoxy asphalt concrete and steel plate composite structure under different curing times of the epoxy resin adhesive, as shown in Table 13. A flowchart for the preparation of the composite specimens is shown in Figure 3.

The interlayer bonding performance of the samples is measured using the PosiTest AT-A digital display automatic control pull-out instrument (Defelsko Corporation, Ogdensburg, New York, NY, USA.). The testing procedure is as follows:

1. Three-Layer Structure (“Steel Plate + Epoxy Zinc-Rich Primer + Epoxy Resin Bonding Layer”)

The composite specimen consists of a three-layer structure: “steel plate + epoxy zinc-rich primer + epoxy resin bonding layer”. The epoxy resin bonding layer is applied to the surface of the specimen at a coating rate of 0.6 kg/m^2^. The specimen is then cured under the temperature conditions specified in Table 12 for different periods, forming the three-layer structure. After curing, the pull-out head is adhered to the specimen using AB adhesive. Once the AB adhesive has fully cured, the area around the pull-out head is cut to the surface of the steel plate. The specimen is then placed at 25 °C for 24 h before measuring the pull-out strength.

2. Four-Layer Structure (“Steel Plate + Epoxy Zinc-Rich Primer + Epoxy Resin Bonding Layer + Epoxy Asphalt Mixture”)

**Figure 4:**
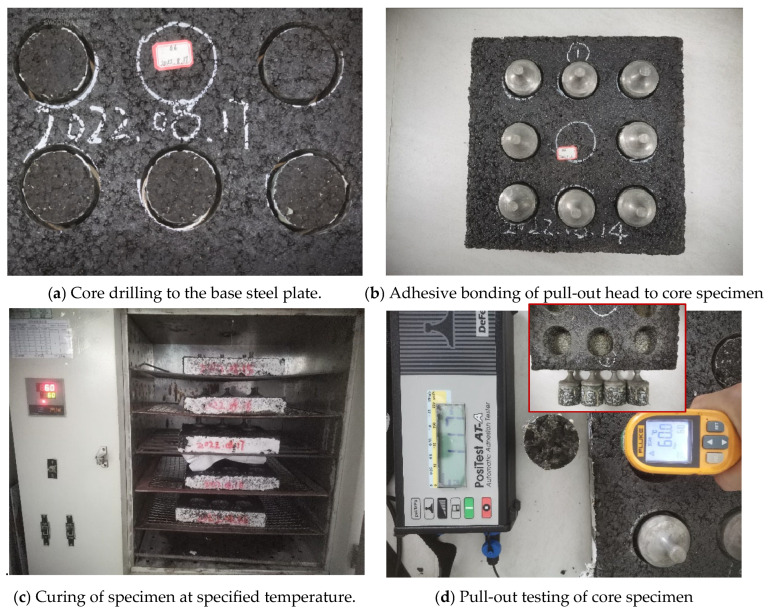
Flowchart of composite specimen pull-out test.

The epoxy resin bonding layer is applied at 0.6 kg/m^2^ on the surface of the specimen. The three-layer structure specimen is exposed to outdoor conditions for a specified number of days and then placed into a rut mold. Hot-mix asphalt is compacted on top of the specimen, forming the four-layer structure. After the standard curing of the epoxy asphalt concrete composite specimen, the surface of the epoxy asphalt concrete is ground with a grinding wheel, cleaned, and air-dried. Core sampling is then performed at the selected test points, drilling to the interface between the paving layer and the steel plate. The pull-out head is then adhered to the specimen with AB adhesive. After the adhesive fully cures, a pull-out test is conducted under stress-control mode (constant loading rate of 150 N/s) at both 23 °C and 60 °C. The test continues until either cohesive failure or adhesive failure occurs in the paving layer material. The measured value reflects the overall pull-out bonding strength of the composite specimen. The flowchart for the pull-out test of the composite specimens is shown in Figure 4.

### 4.2. Experimental Design of Tack-Free Time of Epoxy Resin Adhesive

After the application of the epoxy resin adhesive, it is essential to allow for a curing period before proceeding with the construction of the overlay layer. The adhesive must reach a tack-free state before further construction can take place. If this step is skipped, the construction machinery may experience significant wheel adhesion, which can severely affect both the interlayer bonding performance and the overall stability of the overlay layer. Therefore, experimental analysis was conducted to evaluate the tack-free time of the epoxy resin adhesive under different application rates and temperatures for this project.

**Figure 5:**
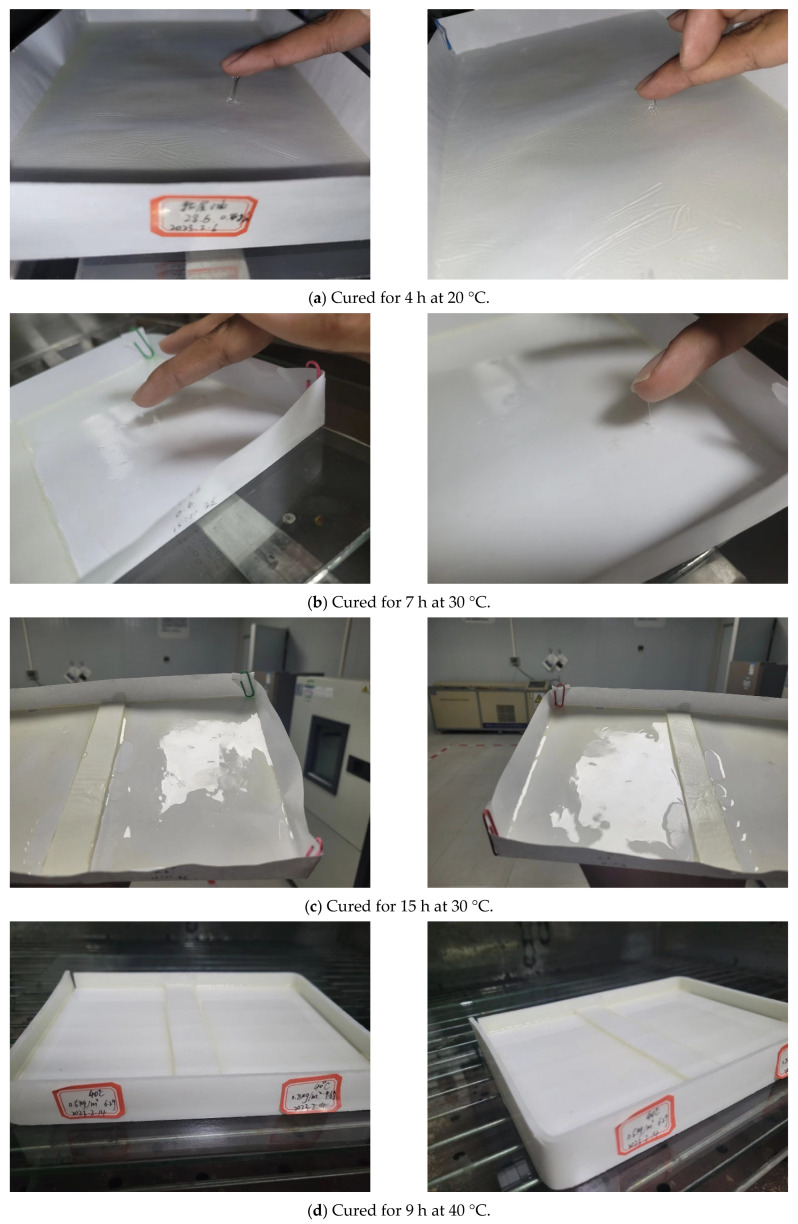

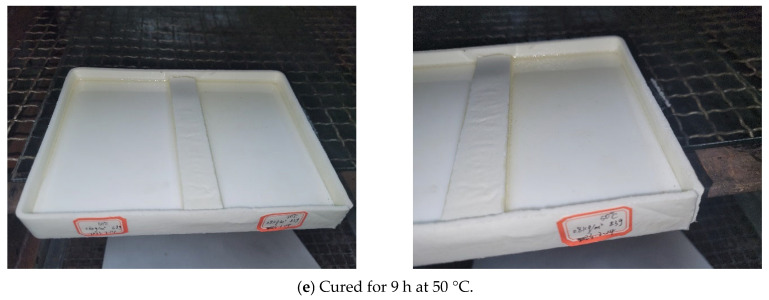
Tack-free state of epoxy resin adhesive under different curing conditions.

To simulate the field conditions, the mixing temperature was controlled at approximately 23 °C, with the ambient temperature maintained at 30 °C. The glass plates were pre-placed in an oven and heated to match the set curing temperature. The epoxy resin adhesive was mixed at a rate of 300 rpm for 3 min continuously. The tack-free time was simulated under conditions of 20 °C, 30 °C, 40 °C, 50 °C, and 60 °C, with two coating thicknesses of 0.6 kg/m^2^ and 0.8 kg/m^2^, as presented in Figure 5, with an average relative humidity of 80%.

## 5. Experimental Results and Analysis of Epoxy Resin Adhesive

### 5.1. Evaluation of Bonding Performance of Epoxy Resin Adhesive

**Figure 6:**
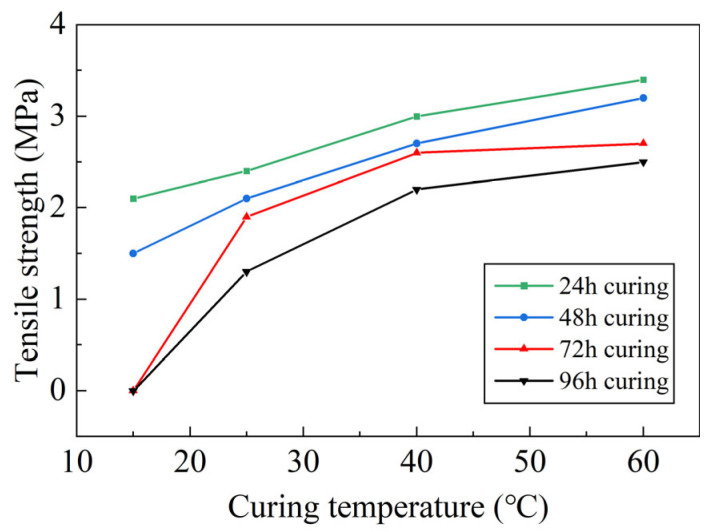
Tensile strength curve of the epoxy resin bonding layer.

The pull-out strength curves between the adhesive layer and the steel plate under various curing temperatures and times are shown in Figure 6. The graph reveals the following key trends and insights:

When the curing period is kept constant, the pull-out strength increases as the curing temperature rises. This suggests that higher curing temperatures accelerate the chemical reactions involved in the curing process, leading to faster polymerization of the adhesive and, consequently, stronger bonding between the adhesive layer and the steel plate. The improved strength at higher temperatures indicates a more complete curing of the adhesive, which enhances its adhesion properties.

Similarly, when the curing temperature is held constant, the pull-out strength improves as the curing time increases. Longer curing times allow for better cross-linking of the adhesive molecules, which contributes to a more robust adhesive bond. The extended curing period gives the adhesive more time to fully develop its bonding properties, resulting in a higher pull-out strength over time.

Provided that the proportion of epoxy resin binder is accurate and the mixture is thoroughly blended, the bonding strength of the waterproof adhesive layer continues to increase as the curing time extends. This is because the correct ratio of resin and hardener ensures optimal chemical interaction, leading to better formation of the polymer network. Proper mixing ensures uniform distribution of the binder, eliminating weak points in the adhesive layer that could compromise its performance.

As the curing time extends, the adhesive layer’s strength grows progressively, especially when the resin mixture is properly proportioned. This reveals a clear pattern: the bonding strength improves with time, but this improvement is most significant when the adhesive has been mixed well and the resin components are in the right proportions. Inadequate mixing or incorrect proportions can delay or hinder the curing process, reducing the adhesive layer’s strength potential.

The data show that both temperature and time play significant roles in determining the final pull-out strength of the adhesive layer. However, these effects are interconnected: temperature accelerates the curing process, while time ensures the complete development of adhesive strength. Additionally, the effectiveness of the adhesive is strongly dependent on accurate mixing and proper binder ratios. Optimizing both curing conditions and adhesive preparation is crucial for achieving the highest bond strength.

After the standard curing of the epoxy asphalt concrete specimens, the pull-out strength of the composite specimen (“steel plate + epoxy zinc-rich primer + epoxy resin waterproof bonding layer + asphalt concrete”) is measured using the PosiTest AT-A pull-out instrument under conditions of 23 °C and 60 °C. This test is conducted to evaluate the overall bonding performance of the epoxy asphalt concrete and steel plate composite structure.

**Figure 7:**
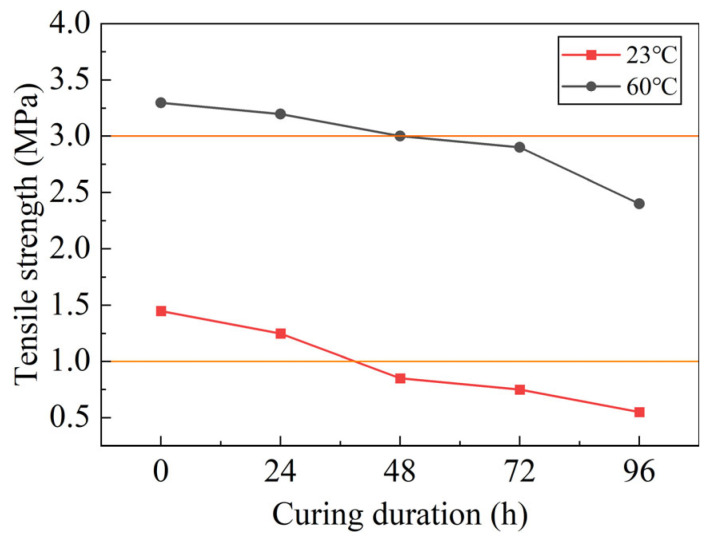
Tensile strength of epoxy resin adhesive at different curing times.

As shown in Figure 7, the pull-out strength at room temperature (23 °C) shows a gradual decrease as the outdoor curing time of the epoxy resin binder increases. After 48 and 72 h of outdoor curing, the pull-out strength approaches the critical value of 3 MPa, and the bonding layer begins to exhibit some degree of failure. After 96 h of outdoor curing, the rate of strength decay accelerates, and the pull-out strength falls well below 3 MPa, with further failure of the bonding layer. This reduction in pull-out strength can be attributed to the aging and degradation of the adhesive layer when exposed to environmental factors over time. Prolonged outdoor curing can result in moisture absorption, temperature fluctuations, and UV degradation, all of which weaken the adhesive’s ability to maintain a strong bond with the steel plate. The epoxy resin binder, while initially strong, may undergo chemical or physical changes, compromising the adhesive properties and leading to a gradual reduction in bonding strength.

The pull-out strength at high temperature (60 °C) also decreases as the outdoor curing time of the epoxy resin binder increases. After 48 h of curing, the pull-out strength drops below 1 MPa, and the bonding layer shows signs of failure. After 72 h of outdoor curing, the pull-out strength is significantly lower than 1 MPa, indicating that the bonding performance of the adhesive under high-temperature conditions is considerably weakened. This trend can be explained by the accelerated curing and potential thermal degradation of the epoxy resin binder at elevated temperatures. The high temperature likely causes the resin to soften or lose its cross-linking ability, making the bond between the resin and the steel plate less stable. In addition, prolonged exposure to high temperatures can induce the thermal expansion or contraction of the materials, generating mechanical stress that further compromises the adhesive layer’s integrity.

When the epoxy resin binder is applied in its liquid state for constructing the epoxy asphalt mixture, and the paving layer and waterproof bonding layer are cured together, the pull-out strength at both 23 °C and 60 °C conditions is the highest. This suggests that optimal curing conditions for both the bonding layer and the asphalt mixture result in the strongest overall bonding performance. The simultaneous curing of both the adhesive and the asphalt mixture ensures that the bonding layer is fully developed and properly bonded to the steel plate before being exposed to environmental factors. This combined curing process enhances the cross-linking of the epoxy resin, promoting a more robust bond between the layers. By curing both layers together, any mismatch in the curing rates between the adhesive and the asphalt mixture is avoided, leading to a more uniform and reliable bond.

**Figure 8:**
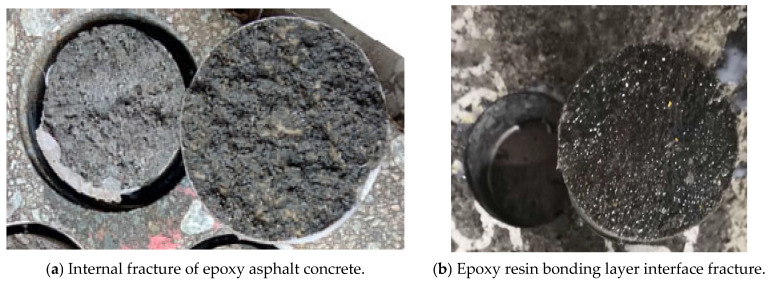
Failure modes of layer interface.

There are two common failure modes observed in the pull-out test of composite pavement layer structures: the internal fracture of the epoxy asphalt concrete and the interface fracture of the epoxy resin bonding layer, as shown in Figure 8. The fracture surface conditions after the pull-out test of the “epoxy asphalt concrete and steel plate” specimens are presented in Table 14, with the statistical results of the fracture mode area proportions shown in Table 15.

The data show that when the epoxy resin binder is uncured or has undergone 24 h of outdoor curing, the fracture surface in the 23 °C pull-out test mainly occurs within the concrete itself, without failure at the interface between the bonding layer and the epoxy asphalt concrete. This indicates that the epoxy resin waterproof bonding layer forms a strong bond with the epoxy asphalt concrete at room temperature under these conditions. The relatively short curing time means that the epoxy resin has not undergone significant aging or degradation, allowing it to maintain its optimal adhesive properties. Therefore, the bond remains robust, and fractures occur within the concrete, not at the interface.

However, when the epoxy resin binder undergoes 48 h, 72 h, or 96 h of outdoor curing, the pull-out test at 23 °C shows that the fracture surface predominantly occurs at the interface between the bonding layer and the asphalt concrete. This suggests that after 48 h of outdoor curing, the bond strength of the epoxy resin bonding layer starts to degrade. The prolonged curing leads to the aging of the resin, which likely alters its chemical structure, reducing its ability to maintain a strong bond with the asphalt concrete at room temperature. Even though the pull-out strength under 48 h of outdoor curing is still above 3 MPa, the fact that the failure occurs at the interface rather than within the concrete suggests that the adhesive layer has become more brittle or less flexible, increasing the risk of inter-layer delamination.

When the epoxy resin binder is uncured or has undergone 24 h of outdoor curing, the fracture surface in the 60 °C pull-out test occurs primarily within the concrete, not at the bonding layer–asphalt concrete interface. This indicates that the bond between the epoxy resin bonding layer and asphalt concrete is still strong even at elevated temperatures. The relatively short curing time prevents the resin from undergoing significant thermal degradation, so the bond remains intact at high temperatures.

Nevertheless, after 48 h, 72 h, or 96 h of outdoor curing, the fracture surface in the 60 °C pull-out test mainly occurs at the interface between the bonding layer and the asphalt concrete, indicating that the bond strength of the epoxy resin bonding layer at elevated temperatures deteriorates after extended outdoor curing. The prolonged curing leads to aging of the resin, which makes it less resistant to high temperatures. The resin likely softens or loses its flexibility, weakening the bond between the layers. This is particularly concerning because, during summer months, the temperature of the steel bridge deck can reach up to 70 °C, and under these conditions, the epoxy resin bonding layer may fail to maintain a strong bond, significantly increasing the risk of delamination between the layers.

As the curing time of the epoxy resin binder increases, its ability to maintain a strong bond with the asphalt concrete decreases. This occurs for both room-temperature (23 °C) and high-temperature (60 °C) conditions. The initially strong bond degrades as the resin undergoes chemical aging during prolonged curing. This aging process likely alters the resin’s molecular structure, making it more brittle or less flexible, and reducing its effectiveness as a bonding agent. This results in failure occurring at the bonding layer interface, rather than within the concrete itself, as the bond is no longer capable of withstanding the applied stresses.

The high temperatures during the summer, combined with prolonged exposure to outdoor curing, accelerate the aging and degradation of the epoxy resin bonding layer. In the case of prolonged outdoor curing, the resin undergoes changes that make it more vulnerable to failure at both room temperature and elevated temperatures. This explains the observed interface failure after extended curing, especially when subjected to high temperatures (60 °C). The combination of aging and heat weakens the resin’s adhesive strength, increasing the likelihood of delamination between the layers, which poses a risk to the structural integrity of the composite system.

The results indicate a higher risk of delamination as the epoxy resin bonding layer loses its ability to maintain a strong bond with the asphalt concrete, particularly after extended outdoor curing. This is a critical concern for composite systems exposed to environmental conditions, especially in high-temperature environments where the adhesive layer’s strength is compromised. The observed failures at the interface between the bonding layer and the asphalt concrete, rather than within the concrete, underline the importance of optimizing the curing process to ensure long-term durability, especially in structures subjected to extreme heat.

### 5.2. Analysis of Tack-Free Time of Epoxy Resin Adhesive

**Figure 9:**
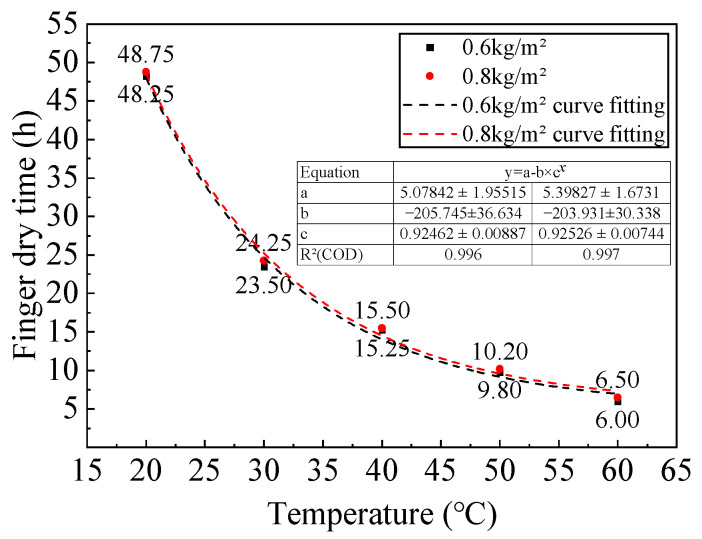
Variation curve of tack-free time of epoxy resin adhesive layer oil under different spraying amounts and temperature conditions.

The state of the epoxy resin adhesive was determined by a finger touch to assess its tack-free status, and the tack-free time was then determined. The experimental results are shown in Table 16 and Figure 9.

Under different temperature conditions, the tack-free time for the two application rates, 0.6 kg/m^2^ and 0.8 kg/m^2^, is quite similar. This indicates that, for both application rates, the primary factor affecting the tack-free time is temperature, with no significant difference between the two application rates.

Based on the fitting curves for tack-free time under various application rates and temperatures, it is evident that the tack-free time has a good linear relationship with the curing temperature, with a goodness of fit of no less than 0.996.

The tack-free time obtained from the experiment differs from the curing time in the design documents, with a difference ranging from 1 to 12 h. The reason for this discrepancy lies in the dynamic range of temperatures at the construction site and the inability to simulate the effect of gusty winds at the site on the tack-free time in an indoor environment.

In previous projects, the conventional practice for applying epoxy resin adhesive was to wait until the following day to lay the epoxy asphalt concrete, with a typical construction cycle of three days. Most epoxy resin adhesive applications were completed in the afternoon or evening. If the adhesive was applied after the hot afternoon period, it needed to undergo curing during the following day’s high-temperature period. However, curing during the night-time low-temperature period was not as effective for achieving a tack-free state. In this project, the efficiency of epoxy resin adhesive application was improved by completing the work before 12:00 p.m. on the same day. With this approach, after the application, curing occurs during the high-temperature period of the day, and by the time construction resumes the next day, the adhesive layer has already undergone at least 19 h of curing. By reducing the curing time during the low-temperature night-time period, the overall construction cycle for the overlay layer can be shortened to two days.

Table 17 presents the recommended curing days for the epoxy resin adhesive under different temperature conditions. For temperatures between 40 and 50 °C, the curing time is 12 h, with a bonding effectiveness period of 36 h and an effective construction period of 36 h. For 30~40 °C, curing takes 24, with the bonding effectiveness lasting 48 h and the effective construction period also 48 h. At 20~30 °C, the curing time remains at 24 h, but the bonding effectiveness extends to 72 h, with the effective construction period at 48 h. For temperatures between 10 and 20 °C, the curing time increases to 48 h, with a bonding effectiveness period of 144 h and an effective construction period of 96 h. This table serves as a critical guideline for determining the optimal curing time and construction window based on temperature conditions, ensuring that the adhesive maintains strong bonding performance and stability. It helps in managing construction schedules, minimizing the risk of bond failure, and enhancing the overall efficiency of the paving process by providing precise curing and application timelines.

## 6. Conclusions

A systematic experimental study was conducted to investigate the effect of curing time on the bond waterproofing layer of steel bridge deck paving, providing technical support for engineering implementation. The analysis and summary are as follows:

(1) Under the same curing period, the tensile bond strength between the waterproof bonding layer and the steel plate increases with higher curing temperatures. Similarly, when curing temperature is held constant, the tensile bond strength increases with longer curing times.
(2) When epoxy asphalt mixture is applied without curing the epoxy resin bonding layer under liquid-state conditions, the tensile bond strength is maximal at both 23 °C and 60 °C. This suggests that continuous construction involving “epoxy resin binder coating + epoxy asphalt concrete paving” is feasible for interlayer bonding. The next step could involve addressing construction organization and equipment issues to explore the integrated construction process of epoxy asphalt paving and coating application.
(3) With 24 h of curing for the epoxy resin bonding layer, epoxy asphalt mixtures applied under 23 °C and 60 °C conditions show sufficient tensile strength, meeting design requirements and ensuring good interlayer bonding performance.
(4) After 48 h of curing, the epoxy resin bonding layer applied to the epoxy asphalt mixture shows tensile strength that meets the requirements at 23 °C, but the fracture surface primarily occurs at the bonding layer interface. At room temperature, there is a risk of delamination between the layers, while at 60 °C, the tensile strength is less than 1 MPa, resulting in the failure of interlayer bonding.
(5) After 72 to 96 h of curing, the tensile bond strength of the epoxy resin bonding layer applied to the epoxy asphalt mixture at both 23 °C and 60 °C no longer meets the design requirements, as the bonding layer’s adhesive properties have been lost.
(6) The tack-free time of the epoxy resin adhesive decreases with elevated curing temperatures or extended durations. Under ambient conditions, a 48–72 h curing period achieves the optimal balance between bond strength development and construction workability (avoiding equipment adhesion).

Future research should focus on optimizing the curing process to improve the performance and durability of the bonding layer. This could involve the development of advanced curing techniques or additives to maintain the adhesive strength over extended periods, especially under varying environmental conditions. Additionally, further investigation into the integration of epoxy asphalt application, curing processes, and construction equipment is essential to streamline the construction process, reduce risks of delamination, and enhance overall structural integrity. Further field tests and long-term monitoring will also be required to validate the effectiveness of these proposed techniques in real-world applications. Future research should incorporate microscopic imaging techniques, such as SEM, EDS, or AFM, to investigate the adhesive bonding mechanisms at the interface and further justify the role of chemical adhesion in bonding performance. Future research should focus on identifying and optimizing the most effective epoxy resin compositions, including variations in resin formulations and substrate conditions, to improve bonding performance.

## Figures and Tables

**Table 1:** Physical properties of epoxy resin adhesive main agent.

**Test Item**	**Technical Requirements**	**Test Results**	**Test Method**
Viscosity (23 °C, Pa·s)	1000~5000	1380	GB/T12007.4 [49]
Epoxy equivalent	170~200	181	GB/T4612-2008 [50]
Flash point (°C)	130	151	
Specific gravity (23 °C)	1.1~1.3	1.142	
Appearance	Slightly yellow transparent liquid	Slightly yellow transparent liquid	Visual inspection

**Table 2:** Physical properties of epoxy resin curing agent.

**Test Item**	**Technical Requirements**	**Test Results**	**Test Method**
Viscosity (23 °C, Pa·s)	500~1100	610	GB/T12007.4 [49]
Acid value (mg, KOH/g)	130~170	144	GB/T4612-2008 [50]
Flash point (°C)	≥145	167	
Specific gravity (23 °C)	0.8–1.0	0.889	
Appearance	Slightly yellow transparent liquid	Slightly yellow transparent liquid	Visual inspection

**Table 3:** Physical properties and technical indicators of epoxy resin adhesive after curing.

**Test Item**	**Technical Requirements**	**Test Results**	**Test Method**
Component A/B weight ratio	50/50	50/50	--
Tensile strength (23 °C, MPa)	≥3.0	4.84	GB/T 528-2009 [51]
Fracture elongation (23 °C, %)	≥100	334	
Bonding strength with steel plate (23 °C, MPa)	≥3.0	3.84	(JTG/T3364-02-2019) [52]
Bonding strength with steel plate (60 °C, MPa)	≥1.0	1.19	

Note: (JTG/T3364-02-2019) refers to “highway steel bridge deck pavement design and construction technical specifications”.

**Table 4:** Physical properties and technical indicators of epoxy resin binder base agent.

**Test Item**	**Technical Requirements**	**Test Results**	**Test Method**
Viscosity (23 °C, Pa·s)	1000~5000	1750	GB/T 22314-2008 [53]
Specific gravity (23 °C)	1.1~1.2	1.132	
Epoxy equivalent	190~220	204	
Flash point (°C)	>220	245	
Appearance	Light-yellow transparent liquid	Light-yellow transparent liquid	Visual inspection

**Table 5:** Physical properties and technical indicators of epoxy resin binder curing agent.

**Test Item**	**Technical Requirements**	**Test Results**	**Test Method**
Viscosity (23 °C, Pa·s)	100~800	295	GB/T 22314-2008 [53]
Specific gravity (23 °C)	0.8~1.0	0.856	
Acid value (mg, KOH/g)	150~200	165	
Flash point (°C)	>145	177	
Appearance	Pale yellow-brown liquid	Pale yellow-brown liquid	Visual inspection

**Table 6:** Technical indicators of epoxy resin binder after curing.

**Test Item**	**Technical Requirements**	**Test Results**	**Test Method**
Weight ratio (base agent/curing agent)	56/44	56/44	--
Tensile strength (23 °C, MPa)	≥3.0	5.25	GB/T 528-2009 [51]
Fracture elongation (23 °C, %)	≥100	152	

Note: Experimental values after 4 days of curing in a 60 °C oven.

**Table 7:** Technical specifications of grade A 70# petroleum asphalt.

**Test Item**	**Technical Requirements**	**Test Results**	**Test Method**
Penetration (25 °C, 100 g, 5 s, 0.1 mm)	60~80	63	JTG E40-2007 [54]
Penetration index (PI)	−1.5~1.0	1.12	
Softening point TR&B (°C)	≥47	47.5	
Ductility (10 °C, 5 cm/min, cm)	≥20	21	
Ductility (15 °C, 5 cm/min, cm)	≥100	>100	
Wax content (distillation method, %)	≤2.0	1.72	
Flash point (°C)	≥260	344	
Solubility (%)	≥99.5	99.8	
Density (15 °C, g/cm^3^)	≥1.000	1.037	
Dynamic viscosity at 60 °C (Pa·s)	≥180	201	
Residual after RTFOT			
Mass change (%)	≤±0.8	−0.078	JTG E40-2007 [54]
Residual penetration ratio (25 °C, %)	≥61	66.7	
Residual ductility (10 °C, cm)	≥6	6.6	

**Table 8:** Technical specifications of coarse aggregate.

**Test Item**	**Technical Requirements**	**Test Results**	**Test Method**
Los Angeles abrasion loss (%)	≤16	13.6	JTG E40-2007 [54]
Polished stone value (BPN)	≥42	44	
Content of needle-like particles (%)	≤5	3.4	
Crushing value (%)	≤15	14.6	
Adhesion to asphalt (grade)	≥4	5	
Water absorption rate (%)	≤1.5	0.57	
Apparent relative density	≥2.70	2.940	
Durability (%)	≤5	3	
Soft stone content (%)	≤1	0	
Content of particles < 0.075 mm (washed method, %)	≤0.8	0.3	

**Table 9:** Technical specifications of fine aggregate.

**Test Item**	**Unit**	**Technical Requirements**	**Test Results**	**Test Method**
Apparent relative density	-	≥2.70	3.004	JTG E42-2005 [55]
Durability (part > 0.3 mm)	%	≤5	4	
Sand equivalent	%	≥70	78	
Methylene blue value	g/kg	≤2.5	0.8	
Angularity (flow time)	s	≥30	37.7	

**Table 10:** Technical performance indicators of mineral powder.

**Test Item**		**Technical Requirements**	**Test Results**	**Test Method**
Apparent relative density		≥2.50	2.756	JTG E42-2005 [55]
Appearance		No agglomerates or clumping of particles	No agglomerates or clumping of particles	
Moisture content (%)		≤1	0.1	
Passing rate (%)	0.6 mm	100	100	
	0.15 mm	90~100	99.8	
	0.075 mm	85~100	90.7	
Hydrophilicity coefficient		≤1	0.56	
Plasticity index (%)		≤4	3	
Stability		Non-altering	Non-altering	

**Table 11:** Test performance indicators of EA10 lower-layer mixture.

**Test Item**		**Unit**	**Technical Requirements**	**Test Results**
Bulk relative density		/	/	2.586
Theoretical maximum relative density		/	/	2.615
Void content		%	0~3	1.1
Marshall stability (60 °C)	Uncured specimen	kN	≥5.4	15.95
	Cured specimen	kN	≥40	72.31
Marshall flow value (60 °C)	Uncured specimen	mm	3.0~6.0	5.77
	Cured specimen	mm	3.0~6.0	36.6
Aggregate void content		%	/	15.3
Asphalt saturation		%	/	89.7
Effective thickness of asphalt film		μm	/	6.267
Dynamic stability (70 °C)		cycle/mm	≥10,000	90,000.4
Freeze–thaw splitting strength ratio		%	≥90	92.6
Residual stability		%	≥90	91.8
Flexural limit strain (−10 °C)		/	≥3 × 10^−3^	4.1 × 10^−3^
Impact toughness (15 °C)		N×mm	≥3000	3425.8

**Table 12:** Bonding performance test scheme of steel plate and epoxy resin adhesive.

**Curing Temperature (°C)**	**Curing Time** **(h)**	**Coating Amount of Adhesive Layer Oil (kg/m^2^)**	**Pull-Off Test Temperature (°C)**
15	24, 48, 72, 96	0.6	23
25			
40			
60			

**Table 13:** Bonding performance test scheme of steel plate and epoxy asphalt concrete.

**Sample Number**	**Curing Time** **(h)**	**Coating Amount of Adhesive Layer Oil (kg/m^2^)**	**Pull-Off Test Temperature (°C)**
1	0	0.6	23, 60
2	24		
3	48		
4	72		
5	96		

**Table 14:** Fracture surface condition of “epoxy asphalt concrete + epoxy resin bonding layer + steel plate” specimens.

**Outdoor Curing Time ** **(h)**	**Sample Number**	**Pull-Out Failure Interface** **at 23 °C**	**Pull-Out Failure Interface** **at 60 °C**
0	1-1	100%C/Y	100%C
	1-2	100%C/Y	100%C
	1-3	100%C/Y	100%C
	1-4	100%C/Y	100%C
24	2-1	100%C/Y	100%C
	2-2	100%C/Y	100%C
	2-3	100%C/Y	100%C
	2-4	100%C/Y	100%C
48	3-1	100%B/C	100%B/C
	3-2	100%B/C	100%B/C
	3-3	100%B/C	100%B/C
	3-4	100%B/C	100%B/C
72	4-1	100%B/C	100%B/C
	4-2	100%B/C	100%B/C
	4-3	100%B/C	100%B/C
	4-4	100%B/C	100%B/C
96	5-1	100%B/C	100%B/C
	5-2	100%B/C	100%B/C
	5-3	100%B/C	100%B/C
	5-4	100%B/C	100%B/C

Note: B—epoxy resin bonding layer, C—epoxy asphalt concrete paving layer, Y—adhesive, B/C—adhesive failure between bonding layer and substrate (steel plate).

**Table 15:** Fracture surface results statistics of “epoxy asphalt concrete + epoxy resin bonding layer + steel plate” specimens.

**Outdoor Curing Time ** **(h)**	**Internal Fracture of Paving Layer (%)**	**Adhesive Layer Interface Fracture (%)**
0	100	0
24	100	0
48	0	100
72	0	100
96	0	100

**Table 16:** Tack-free time of epoxy resin adhesive layer oil under different spraying amounts and temperature conditions.

**Material Name**	**Curing Temperature (°C)**	**Spraying Amount (kg/m^2^)**	**Tack-Free Time (h)**
Epoxy resin adhesive layer oil	20	0.6	48.25
		0.8	48.75
	30	0.6	23.5
		0.8	24.25
	40	0.6	15.25
		0.8	15.5
	50	0.6	9.8
		0.8	10.2
	60	0.6	6
		0.8	6.5

Note: Cured using a high- and low-temperature oven.

**Table 17:** Recommended curing days for epoxy resin adhesive.

**Temperature Condition** **(°C)**	**Curing Time (h)**	**Bonding Effectiveness Period (h)**	**Effective Construction Period (h)**
40~50	12	36	36
30~40	24	48	48
20~30	24	72	48
10~20	48	144	96

## Data Availability

Data are contained within the article.
